# Early cancer diagnosis and community pharmacies: a systematic review

**DOI:** 10.3399/BJGP.2025.0204

**Published:** 2026-03-24

**Authors:** Judit Konya, Rachel Winder, Mary D Carter, Gianni Dongo, David Bearman, Gary A Abel, Ziad A Laklouk, John L Campbell, Richard D Neal, Christopher E Clark

**Affiliations:** 1 Exeter Collaboration for Academic Primary Care (APEx), University of Exeter, Exeter, UK; 2 Community Pharmacy Devon, Exeter, UK; 3 Primary Care and Community Services Directorate, NHS England, London, UK

**Keywords:** cancer, community care, primary health care, systematic review

## Abstract

**Background:**

Patients with symptoms of suspected cancer usually present to general practice; however, they may present to other healthcare providers, such as community pharmacies. The clinical role of pharmacy staff has significantly increased in England in recent years.

**Aim:**

To describe the range of interventions targeting early cancer detection in community pharmacies globally; to summarise the outcomes of these interventions; to report on barriers and facilitators to delivering the interventions and on service users’ and stakeholders’ experiences with such interventions.

**Design and setting:**

Systematic review with narrative synthesis, of international literature, on community pharmacy-based early cancer detection.

**Method:**

Online searches of Medline, CINAHL, Cochrane CENTRAL, PsycINFO, and Embase and UK-based relevant grey literature were performed. Interventions were defined as any intervention, initiative, or programme that focused on community pharmacy-based early cancer detection programmes.

**Results:**

In total, 14 134 titles and abstracts were screened, and 330 full-text publications were reviewed by two independent reviewers. Of these, 52 publications were included in the review. They reported on interventions focusing on early diagnosis of colorectal (*n* = 19), skin (*n* = 8), lung (*n* = 4), cervical (*n* = 3), breast (*n* = 2), head and neck (*n* = 2), and mixed (*n* = 14) cancers. The feasibility and acceptability of such interventions by community pharmacy staff and customers/patients have been demonstrated in the included studies. Studies involving opportunistic identification of customers with suspected cancer symptoms in pharmacies recruited only a few participants.

**Conclusion:**

Robust, large-scale clinical trials are needed to demonstrate cost-effectiveness, delineate and inform the use of relevant clinical outcomes, and to explore arrangements for information sharing between community pharmacy and other healthcare settings.

## How this fits in

Early detection of cancer is crucial in achieving good clinical outcomes for affected patients. Patients with symptoms of possible cancer usually present to general practice in the first instance, but may also present to other healthcare services, such as community pharmacies. Hence, reviewing the available literature on community pharmacy-based early cancer detection approaches is important to guide further policy and service planning with a view to improving the rate of early cancer detection and reducing health inequalities.

## Introduction

Early detection of cancer is associated with improved clinical outcomes for affected patients.^1,2^ Patients most often present to general practice with cancer signs and symptoms.^3^ However, they may present before diagnosis to other healthcare providers such as community pharmacies (‘pharmacies’).^4^ Pharmacies have an emerging role in the clinical assessment of customers.^5–7^ Patients in deprived areas have more difficulties in accessing general practice compared with those from less deprived areas.^8^

The ‘positive pharmacy care law’ shows that in England a higher proportion of the population in deprived areas lives within a 20-min walk to a pharmacy compared with more affluent areas.^9^ Across England, therefore, geographical access to pharmacies is better than access to general practice surgeries.^9,10^

Pharmacies can contribute to the prevention, screening, and early diagnosis of cancer,^11^ and collaborative working partnerships are being explored between general practice and pharmacies.^12^ Patients in deprived areas experience a higher disease burden related to cancer, and they are more likely to be diagnosed at a late stage compared with patients in affluent areas.^13,14^

A systematic review published in 2015 summarised the available evidence on pharmacies and cancer screening and education.^15^ An up-to-date review of the international evidence on pharmacy-based approaches to early cancer detection is now needed to aid future policy development, and which takes into account the growing clinical responsibility of pharmacy staff.^16^ The aim of the current systematic review was to summarise the evidence regarding the current role and future potential of community pharmacies in early cancer diagnosis, with a focus on deprived areas. The objectives were:

- to describe the range of interventions targeting early cancer detection in pharmacies globally;
- to present the clinical and behavioural outcomes of these interventions compared with usual practice;
- to report on service users’ and stakeholders’ experiences with such interventions; and
- to report on barriers and facilitators to the identified interventions.

The study set out to undertake subgroup analysis between approaches according to deprivation to account for the different healthcare needs of the population.

## Method

The methods are described in full in the PROSPERO registered protocol (registration number CRD42023410485) and described in brief here.^17^ The review is reported in accordance with the PRISMA statement.^18^

### Public and patient involvement and engagement

The public and patient involvement and engagement team consisted of two groups: a public advisory and a professional stakeholder group. The latter included community pharmacists. They reflected on the need for the review, provided input into the design of the data-collection tools and the synthesis of the findings, and contributed to the authors’ dissemination plans.

### Eligibility criteria

Eligible to be included were publications from electronic peer-reviewed and UK-based grey literature sources, reporting on pharmacy-based interventions involving pharmacy staff, other stakeholders such as general practice staff or commissioners, and/or service users targeting early cancer detection, published in or after 2015. Interventions were broadly defined as any intervention, initiative, or programme. Publications were excluded from data synthesis if they were ongoing studies, conference abstracts only, or if there were no reported outcomes.

### Search strategy

Search strategies were developed with the help of information specialists at the University of Exeter. Searches were performed in April 2023, and re-run in April 2024. The following databases were searched: Ovid Medline, Embase, APA PsycINFO, Cochrane CENTRAL, CINAHL, and relevant UK-based grey literature websites. The full list of resources and the search strategy are included in Supplementary Box S1.

### Data collection

Titles, abstracts, and full-text articles were screened and reviewed by two independent reviewers using Covidence (Veritas Health Innovation, Melbourne, Australia, https://www.covidence.org/). Eligible publications written in languages other than English were translated with the help of academic colleagues. Disagreements between reviewers were resolved by discussion or by involving a third reviewer.

### Quality assessment

For peer-reviewed publications, quality was assessed using the Mixed Methods Appraisal Tool (MMAT)^19^ (Supplementary Table S1) and grey literature publications were quality assessed using the Authority, Accuracy, Coverage, Objectivity, Date, Significance (AACODS) checklist^20^ (Supplementary Table S2). Publications were included irrespective of the results of their quality assessment.^21^

### Synthesis of results

Owing to the heterogeneity of methods and types of included studies, a narrative synthesis approach was used.^22^ Heterogeneity of included studies precluded the a priori planned meta-analysis.

## Results

**Figure 1.**
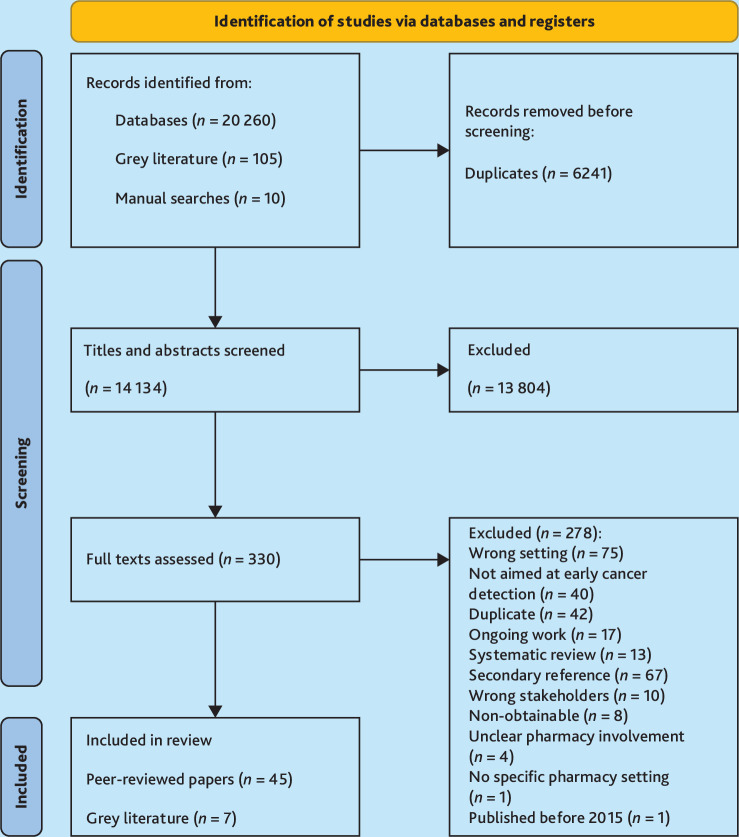
PRISMA flowchart.

In total, 14 134 unique titles and abstracts were screened following de-duplication, with 330 full texts assessed for eligibility and 52 included in the review (Figure 1). The searches were re-run in 2024. The characteristics of the included studies and identified ongoing works are summarised in Supplementary Table S3 and S5.

The 52 included publications reported on interventions focusing on colorectal cancer (CRC, *n* = 19), mixed (*n* = 14), skin (*n* = 8), lung (*n* = 4), cervical (*n* = 3), breast (*n* = 2), and head and neck cancers (*n* = 2). The studies wereconducted in the UK (*n* = 16), Europe (*n* = 20), the US (*n* = 9), and in other countries (*n* = 7). Seventeen ongoing works were further noted (Table 1).

**Table 1.** Included publications and ongoing works by cancer site and country of origin

Site	England	Scotland	Wales	Ireland	UK	US	Spain	Italy	Ghana	Australia	France	Belgium	Switzerland	Norway	North Cyprus	Palestine	Jordan	Turkey	Total
**CRC**	^62,70,73^	^71,72^		^27^	^65^	^23–26,28,40,64^	^31,32,37–39^	^33–36,84^	^68^	^41^	^29^		^30^						28
**Skin**			^48^		^44^	^42,43,78^	^46,47,88^					^49^		^45^				^87^	11
**Lung**	^54,70,73,77^, ^83^	^52,71,72^	^53^	^55^					^68^										11
**Cervical**	^62^						^59,75^	^58,60^	^68^										6
**Breast**	^62,70^					^64^			^68^	^82^						^57^	^56^	^90^	8
**Head and neck**	^50,51^																		2
**Ovarian**	^70^																		1
**Prostate**	^70^								^68^										2
**Testicular**									^68^										1
**Endometrial**									^68^										1
**Upper GI**		^72^																	1
**All**	^4,61,62,66,73,74,79–81,86,89^		^85^		^65,76^				^67,69^						^63^				17
**Total**	26	6	3	2	4	11	10	7	9	2	1	1	1	1	1	1	1	2	

Where two or more cancer types were studied in a publication, and cancer types were specified, the publication is listed multiple times in the table, under the separate cancer sites. References 27, 54, 61, 62, 66, 71, 73 are grey literature. References 74-90 are ongoing studies. CRC = colorectal. GI = gastrointestinal.

Included publications reported on various aspects of early cancer detection: identification of risk factors, risk assessment, symptom awareness, screening, referral process, and diagnosis. In addition, publications that reported on knowledge assessment or education relating to these steps were grouped as assessment of professional and/or patient knowledge, patient education, or professional training (Supplementary Table S4). Findings relating to the first and second study objectives are presented by cancer type, and non-comparable behavioural and clinical outcomes are summarised in Supplementary Table S3. Findings relating to the third and fourth objectives of the study are summarised together, irrespective of the studied cancer types.

### Description of interventions and outcomes

Non-comparable outcomes of the included studies can be found in Supplementary Table S3.

#### Colorectal cancer

Four publications reported on service users’ and primary care practitioners’ perceptions about a proposed pharmacy-based CRC screening (CRCS) programme called PharmFIT, which involved faecal immunochemical test (FIT) distribution.^23–26^ Studies included 30 to 1045 participants (service users and general practice doctors) over 2–6 months.

In four other trials customers were assessed for their eligibility for CRCS and were provided with FIT kits in pharmacies.^27–30^ They included 16–771 participants over 6 weeks to 9 months.

Nine studies reported on pharmacy-based CRCS, as usual practice.^31–39^ They included 74–1 230 683 participants over periods ranging from 8 weeks to 5 years. Main outcomes were: CRCS uptake rates,^31,33,35^ CRC mortality rates,^36^ pharmacies’ adherence to the CRCS,^39^ pharmacists’ perceptions of providing the CRCS,^37^ patients’ satisfaction with the programme,^32^ assessing the effect of different invitation strategies,^38^ improvement of the process by applying failure models.^34^ Comparable clinical outcomes are presented in Table 2.

**Table 2.** Comparable outcomes of included colorectal cancer screening (CRCS) studies

**Study**	**Eligibility rate for programme (approached patients, eligible patients)**	**Eligibility rate for screening (patients signing up for programme, eligible for screening)**	**Participation rate (eligible for screening, kit issued)**	**Completion rate (returned kit, issued kit)**	**Screening test positivity rate (positive kit, issued kit)**	**Diagnostic test positivity rate (positive colonoscopy, positive screening tests)**	**Detection rates**
**Pharmacy-based CRCS**							
Holle *et al* ^28^	5	50	88	100	14	0	—
Flaherty and Farrelly^27^	—	81	100	74	—	—	—
Le Duff *et al* ^29^	—	—	36	36	6	13	—
Ruggli *et al* ^30^	—	97	97	—	7	Incomplete data	Incomplete data
**Usual care is pharmacy-based CCRS**							
Burón^31^	n/a	n/a	—	44	6	—	Low-risk adenoma 9‰; high-risk adenoma 22‰; invasive cancer 3‰
Vives *et al* ^39^	n/a	n/a	40	94	—	—	—
Parente *et al* ^36^	—	Eligible people, *n*: 80 915	—	—	—	—	Cancers detected via screening, *n*: 95
Chiereghin *et al* ^33^	—	—	62^a^	94–97^b^	—	—	—
Mancini *et al* ^35^	—	—	53^c^	—	—	—	—
Stoffel *et al* ^38^	—	—	36	—	—	—	—

Data are % unless otherwise indicated. ^a^62% after, vs prior to pharmacy integration into the screening programme 57%. ^b^In 2 consecutive years. ^c^Response rate: 53% (PCC 52%, mailing 50%). n/a = not applicable. PCC = Primary Care Centre.

Two further publications reported on other approaches to early diagnosis of CRC.^40,41^ They included 42 and 164 participants, over 4 and 8 months, respectively. One compared referral from pharmacies to GPs of patients who were at high risk of CRC by usual practice with a paper-based CRC-risk assessment tool.^41^ The other reported on an educational intervention where participants eligible for CRCS were identified and referred to their GP for screening.^40^

In one study, half of the GPs surveyed (*n* = 3) thought GPs should provide CRCS counselling,^29^ and in three studies patients and primary care practitioners indicated preference for GPs to communicate abnormal FIT test results to patients, rather than pharmacists.^23–25^

Where outcomes of pharmacy-based CRCS or rate of referral to GPs were compared with usual or other practices, the uptake of CRCS via pharmacy-based approaches were reported to be higher,^27,29,33^ and staff more often referred patients with CRC symptoms to their GP^41^ as part of the interventions.

#### Skin cancer

Eight studies reported on skin cancer-related pharmacy-based interventions:^42–49^ skin assessment in the pharmacy with subsequent signposting to their GP or a dermatologist,^47^ educational intervention and dermatology referral,^49^ teledermatology assessment^44–46^ in case skin abnormalities were found (Table 3). They included between 56 and 15 777 customers over periods of 3 weeks to 46 months. One intervention aimed to identify skin cancer risk factors,^48^ and two interventions were symptom awareness campaigns.^42,43^

**Table 3.** Comparable outcomes of included skin cancer studies: teledermatology

**Study**	**Lesions assessed,** ***n***	**Scan outcomes, % of number of lesions assessed**	**Follow-ups recommended, *n* (%)**	**Follow-up available,** ***n***	**% of diagnoses of available follow-up scans**
**Mendonca** ***et al*** ^46^	225	53.8% benign, 23.1% NMSC precursor, 16.4% dubious, 5.8% NMSC, 0.9% melanoma	n/a	n/a	n/a
**Kirkdale** ***et al*** ^44^	9980	88.7% no follow-up required: 57.4% normal, 22.4% SK, 6.1% potential sun damage, 1.7% other normal lesion, 1.1% normal with atypical characteristics	1118 (11.3)	757	44.3% normal, 26.0% sun damage, 11.0% BCC, 6.6% other, 6.2% melanoma, 3.0% SK, 2.5% atypical, 0.4% SCC
**Kjome** ***et al*** ^45^	25 836	92.1% no follow-up required: 88.3% normal, 2.4% benign skin damage, 1.4% other benign skin condition	2033 (7.9)	1793	51.0% normal, 2.5% sun damage, 23.0% other skin condition, 8.6% cancer or pre-cancer melanoma, 9.3% other skin cancer, 5.6% no outcome

BCC = basal cell carcinoma. n/a = not applicable. NMSC = non-melanoma skin cancer. SCC = squamous cell carcinoma. SK = seborrheic keratosis.

In one publication the authors estimated that provision of the service would be cost-saving for the whole healthcare system in the UK,^44^ based on GP appointments being freed up. In a different study from Norway, the service contributed to up to 4.1% of annual national melanoma diagnoses.^45^

#### Head and neck cancers

Two small studies reported on patient and pharmacists’ views on pharmacy-based detection using clinical decision support tools, and referrals.^50,51^ They included 13–17 participants over 14 months, where duration was reported. Patients with head and neck cancers perceived the use of risk-assessment tools as beneficial, while pharmacists were unaware of the existence of such tools.^50,51^ Pharmacists felt they had limited skills for holistic assessment.^51^ They were keen to be involved in referral pathways.^51^

#### Lung cancer

Four studies reported on interventions targeting lung cancer.^52–55^ They included 13–60 participants over 3 to 13 months. Two studies evaluated direct access chest X-ray referrals from pharmacies.^53,54^ There were no lung cancer diagnoses made. One study explored ways to increase engagement with low-dose computer tomography (LDCT) referral including the role of pharmacies in biomarker testing,^52^ where the biomarker blood test refers to a test that identifies individuals who are at high risk of lung cancer. Pharmacists suggested using drop-in clinics in pharmacies, and members of the public indicated their preference for discussing the biomarker blood test and LDCT with pharmacy staff, or dropping the completed test off at pharmacies.^52^ One study showed low awareness of rapid-access lung clinics among pharmacists in Ireland.^55^

#### Breast cancer

Two questionnaire studies evaluated pharmacists’ attitudes towards breast cancer health promotion.^56,57^ They included 200 and 602 pharmacists, over 3 months, where data collection duration was reported. Participants’ attitudes towards breast cancer health promotion were positive.

#### Cervical cancer

Two randomised controlled studies reported on approaches to pharmacy-based cervical cancer screening,^58,59^ and one other study assessed the long-term effect of pharmacy-based cervical cancer screening.^60^ They included 1209 to 14 041 participants over 6 to 31 months.

Participation in cervical cancer screening was higher when self-sampling devices were mailed to participants’ homes compared with collection from pharmacies or usual primary care screening.^58^ A follow-up study found worse participation in the next screening round in the pharmacy arm compared with usual screening.^60^ Another study found that participants preferred to collect the self-sampling kit from and return to a primary care clinic or a pharmacy, compared with a mailing approach.^59^

#### Mixed cancers

Fourteen publications reported on interventions targeting multiple cancer types.^4,61–73^ They included 25 to 642 participants over 3–14 months.

Three publications explored the frequency and management of possible red-flag symptoms in pharmacies.^4,71,72^ Patients often managed their initial symptoms by taking over-the-counter (OTC) medication.^71^ Repeat medication purchases were unchallenged by pharmacy staff. Although patients rarely sought advice when purchasing OTC medication, those who did received appropriate advice from pharmacy staff.^72^ The most common presenting red-flag symptom was persistent cough lasting >3 weeks.^4^

One publication reported on a nationwide survey study exploring pharmacists’ attitudes to providing support for patients presenting with potential signs or symptoms of cancer. The study found that most responders encourage patients to respond to cancer signs and symptoms, and encourage them to participate in CRCS.^65^

One publication reported on patient adherence to screening programme recommendations after annual health and wellness pharmacy visits.^64^ It showed that, when pharmacists scheduled screening appointments for patients, adherence to the screening recommendation was higher compared with when patients arranged their appointments for themselves.

One publication reported on the acceptability of an online symptom-based cancer risk tool.^70^ Most participants felt that the tool should be completed with the help of healthcare professionals, such as a pharmacists. Four publications reported on pharmacists’ knowledge of cancer and their perceptions of cancer health promotion.^63,67–69^ Most participants agreed that cancer health promotion was an important part of their role.^63,69^ One study reported that knowledge of cancer increased following an online training course.^67^ Two publications reported on the impact of pharmacy-based complex health awareness programmes.^61,62^ Most participants reported that they acquired new information about cancer. One study reported on a programme in which patients received a GP referral card if they presented with potential cancer symptoms.^66^ The most frequent red-flag symptoms were related to potential skin cancer.

A large-scale cancer awareness programme investigated five different pharmacy-based approaches.^73^ These included direct chest X-ray referrals. Participating pharmacy staff reported that the training they received as part of the programme increased their understanding of cancer and screening processes. Customers reported that pharmacists should raise awareness of cancer. Customers felt they had confidence in pharmacy staff communicating health messages.

### Participant experiences of pharmacy-based interventions targeting early cancer diagnosis

#### Acceptability

Interventions were deemed acceptable to service users, pharmacy staff, and other stakeholders.^23,25,26,45,53,54,62,66^ Services were perceived positively by service users^32,43^ and staff members.^43,61^ Service users and pharmacy staff were satisfied with the programmes.^37,45,53^ The information provided to service users by pharmacy staff was considered to be clear^32^ and of high quality.^43,48^ In four publications there were concerns raised by GPs and patients, about participation of pharmacies in CRCS.^23–25,29^

#### Pharmacy as a venue

Pharmacies were thought to be a suitable venue to provide services related to early cancer detection.^25,45,48,61^ Pharmacies were viewed as easily accessible.^25,27,32,50–53^ Privacy in pharmacies was raised as a concern by some patients^25,72^ or by pharmacy staff,^41^ whereas others saw the availability of confidential space as a facilitator.^50,62^

#### Role of pharmacy

The quality of communication between pharmacies and other healthcare providers varied.^53,54^ Concerns regarding communication between general practice and pharmacies were raised,^23,25,51,54,73^ including no formal referral pathways^51^ or shared electronic data capture.^54,55^ One study highlighted good relationships between pharmacies and general practice.^51^ Using cancer risk assessment tools in pharmacies was seen as beneficial;^50,51,70^ however, two publications reported on pharmacists being unaware of head and neck cancer diagnostic tools.^50,51^

#### Role of pharmacy staff

Most service users were comfortable with the pharmacists’ role in such programmes.^25,48^ The role of pharmacists was generally positively perceived by service users.^28,47,66,73^ Some primary care professionals^23^ and patients^50^ felt pharmacists would need training to fulfil their roles in early cancer detection-related activities. Staff members expressed the view that staff other than pharmacists should also be able to provide these services.^27,61,66^ There was some concern regarding availability of pharmacists^53^ by patients^50^ and participating staff,^73^ including a barrier that locum pharmacists were unable to refer.^73^ Some studies reported customers had limited understanding of the advanced healthcare role of pharmacy staff,^50,57,63,72^ whereas others reported patients choosing to seek advice owing to their perceived expertise.^71^

Pharmacists expressed their belief that their role in cancer health promotion was important^63,69^ and counselling skills training was essential to be able to participate in early cancer detection programmes.^73^ In one publication, pharmacists expressed their concerns about having limited skills for holistic patient assessment.^50^

Pharmacy staff often reported a disruption in workflow^28,41^ or time constraints.^43,51,61,62^ Patients perceived pharmacies to be too busy to provide advice about symptoms.^50,72^ The need to fund pharmacies appropriately for their participation in early cancer detection initiatives was highlighted by participants.^28,37,52,53^

#### Public awareness

Some patients were unclear about the provided referral service.^54^ The need for increased awareness of the pharmacy-based programmes targeting early cancer detection within the community was emphasised.^27,52,53,61^

### Barriers and facilitators

Five studies reported on barriers to cancer health promotion, of which two focused on breast cancer.^56,57,63,68,69^ Pharmacists ranked barriers that were provided in the surveys, and the most highly perceived barriers were lack of:

- established guidelines on cancer screening;^68^
- cancer educational materials;^69^
- interest in oncology;^63^
- breast cancer educational materials;^56^ and
- staff.^57^

Barriers and facilitators emerging from the other studies (not related to cancer health promotion) are summarised in Boxes 1 and 2.

**Box 1.** Barriers reported in the included studies

Barriers	Customer level	Pharmacy staff level	Pharmacy level	System level
**Patient/customer reported**	Lack of time^28,52^ Lack of interest^28^ Low symptom awareness and recognition^72^ Reluctance to discuss symptoms^72^	Lack of awareness of pharmacy staff’s role in early cancer detection^53,72^ Age and gender of counter staff^72^ Capacity of staff^53^	Lack of privacy^25,72^ Variation in the desired level of pharmacy input^25^ Pharmacy too busy^72^ Low awareness of services offered in pharmacies^53^	
**Pharmacy staff reported**	Fatalism by patients^27^ Forgetting to do the test^27^ Restricted eligibility criteria^27,54,61^ Lengthy process of intervention^43^ Lack of time^61^ Having been affected by cancer^61^ Reluctance to discuss symptoms^61^ Clarity on project^54^ Urgent referrals causing distress to patients^55^	Pharmacist- only recruitment^27^ Time-consuming project training^41,43^ Lack of time^43,51,61,62^ Change in staffing^61^ Lack of staff^73^ Locums are unable to refer^73^ Frequent use of locums^51^ Knowledge gap^51^ Limited role^55^ Limited scope^55^ Fatigue from repeat patient presentations^55^ ‘Double-edge sword‘ of knowing the patient^55^	Disruption in workflow^28,41^ Lack of compensation^28^ Competing initiatives^61^ Accessing training information challenging^73^ Accessing good-quality information time consuming^73^ Engagement with large pharmacy chains^73^ Unavailability of consultation space^62^ Nature of over-the-counter consults^51^ Project set-up^54^	Unsuitability of test^27^ Lack of project advertising^27^ Customers do not understand the project^61^ Poor communication between pharmacies and other healthcare settings^51,54,73^ Lack of referral pathways^51^ Lack of integrated data flow^54^ Pressures on the healthcare system^55^
**Other stakeholder reported**				Over-screening^23^ Workflow ownership^23^ Care coordination^23^

**Box 2.** Facilitators reported in the included studies

Facilitators	Customer level	Pharmacy staff level	Pharmacy level	System level
**Patient/customer reported**		Listening skills^28^ Ability to explain concepts^28^ Ability to answer questions^28^ Good advice provided^28^ Friendly^28^ Helpful^28^ Approachability^53^ Trust^52^	Easy access^25,53^ Free access^25^	Fast-track nature of referrals^53^
**Pharmacy staff reported**	Having been affected by cancer^61^ Staff being seen as ‘peers’^62^ Already established good relationship between customers and pharmacy staff^62^	In-person interactions^27^ Approachability^27^ Trust^27^ Counselling training vital^73^ Face-to-face training^73^ More than one team being present at training^73^ Regular engagement with staff regarding the project^62^ Being considered knowledgeable by customers^62^	Good access^27^ Being in the community^62^ Privacy^62^	

### UK-based literature

The included literature from the UK (*n* = 16) reported on studies of skin cancer,^44,48^ mixed cancers,^4,61,62,65,66,70–73^ head and neck cancer,^50,51^ and lung cancer.^52–54^ Six of these reports were from the grey literature. The UK-based publications reported on diverse outcomes. Identification of red-flag symptoms in pharmacies showed that the most frequent red-flag symptoms were persistent cough lasting >3 weeks^4^ and skin changes.^66^ Patients manage their suspected cancer symptoms by OTC medication without significant interaction with pharmacy staff.^71,72^ Where they discussed their symptoms, appropriate advice was provided; however, frequent purchases were not challenged by staff members.^72^ Most pharmacists encourage patients to act on potential cancer symptoms and help them to decide on participation in CRCS.^65^ Some included studies showed that cancer risk assessment tools would have a role to play in pharmacy-based early cancer detection.^50,51,70^ Where patients were directly referred for chest X-ray with suspected lung cancer symptoms, no cancer was diagnosed^53,54,73^ and the included number of patients was 12–60 over 8–13 months in 9–61 pharmacies. A red-flag referral card scheme was run in 10 pharmacies over 6 months, and 38 service users were given a card.^66^

One large study reported on a UK-wide teledermatology service where customers paid for the assessment that was provided in 50 pharmacies, including the assessment of 9880 scans, and clinical outcome data were provided for 9519 scans and the authors indicated potential substantial cost-savings if the service was extended nationally.^44^

### Publications reporting on interventions where eligible participants were opportunistically identified in pharmacies

Across all publications, where eligible customers for the trialled interventions were opportunistically identified by pharmacy staff (excluding the teledermatology approaches and including publications where the duration of the intervention, number of participating pharmacies, and eligible participants were reported) the numbers of eligible participants were generally low (UK: eight studies recruited 12–5739 participants, over 1–14 months in 6–376 pharmacies; non-UK: seven studies recruited 42–23 024 participants, over 3 weeks to 9 months, in 1–771 pharmacies; average number of participants per pharmacy per month: UK *n* = 0.34, non-UK *n* = 0.86).^4,27,29,30,40,41,47,48,53,54,61,62,64,66,73^

### Ongoing studies (excluded from the review)

Although ongoing studies were excluded from the review, owing to their relevance and for completeness they are summarised separately here. Out of the 17 ongoing studies,^74–90^ 10 were identified as reporting on UK-based interventions^74,76,77,79–81,83,85,86,89^ (Supplementary Table S5). These show that primary care healthcare professionals believe that pharmacy staff are well placed to refer patients directly to screening and early cancer diagnostic services, and that the main barrier was lack of skills or clinical knowledge.^89^ Another report highlighted the need for training.^86^ The online learning resource, ‘Let’s Communicate Cancer’, was assessed positively by pharmacy staff who accessed the training.^81^ Four of 10 people were concerned about discussing their health concerns with pharmacy staff members as they were worried about incorrect clinical decisions.^76^

A study evaluating the uptake of LDCT by opportunistic referrals from various services reported that 15 227 individuals were approached, but none from pharmacies.^83^ Currently there are plans to roll out the red-flag card scheme to more than 1000 pharmacies.^79^ NHS England is conducting a pilot study across four Cancer Alliances in England, where community pharmacy staff are able to refer patients directly to secondary care or diagnostic services with suspected cancer symptoms.^80^

### Deprivation and rurality

Deprivation and rurality of recruitment area and participants were reported heterogeneously. Deprivation was considered by reporting participants’: education level, household income, and insurance status;^24,26^ education level only;^25,40^ education and employment;^28,59^ education and income;^43^ income, education, and employment;^42^ citizenship;^35^ deprivation score index;^39^ Carstairs Index;^44^ and Index of Multiple Deprivation.^48,52,71^ Two publications reported to have recruited to include diverse socioeconomic groups^41^ or from areas of low, medium, and high deprivation.^4^ Five studies reported to have recruited from deprived areas.^53,54,61,62,73^

Rurality of participants was reported as: rural, urban, and suburban;^24,26,72^ rural, urban, not available;^48^ plain, hill, mountain;^35^ rural;^42^ rural, urban, big city;^47^ and urban.^55^ Pharmacy-level rurality was reported as urban, rural, not available;^48^ rural/urban;^43^ urban, town, fringe.^4^ One publication reported to have recruited from a convenience sample of rural and city pharmacies.^66^ In most publications rurality could have only been assumed based on the description of the recruitment area.^23,25,27–29,31,32,37–39,41,53,54,58,61,62,64,70,71^

Deprivation was considered in reporting results in eight publications,^4,24,26,31,39,43,48,71^ and rurality was considered in three of these.^24,26,43^ Outcomes were diverse hence no analysis was feasible. Results are reported in Supplementary Table S3.

### Quality assessment of included publications

The included publications were generally of good quality (Supplementary Tables S1 and S2). Publications met 20–100% of the quality criteria.^91^

## Discussion

### Summary

This systematic review summarised the global literature from the past 10 years on pharmacy-based early cancer detection publications. There was large heterogeneity in the included publications’ countries of origin, cancer sites investigated, early cancer detection interventions, research methods, and outcomes. The included publications reported on approaches focusing on identification of cancer risk factors, awareness of cancer symptoms, cancer screening, referral processes, cancer risk assessment, cancer diagnosis, knowledge assessment, education, and training.

Cancer detection approaches as usual practice differed between countries. Most of the included papers reported on pharmacy-based CRCS (*n* = 17). Four studies from the US explored the views of healthcare professionals and patients on CRCS in pharmacies.^23–26^ In Spain and Italy, pharmacy-based CRCS was usual practice;^31–39^ skin cancer approaches in Spain, Norway, and the UK included teledermatology referrals; however, these have the potential to widen health inequalities as customers paid for the scans themselves.^44–46^ Publications from Ghana, North Cyprus, Jordan, and Palestine focused on knowledge of cancer among pharmacists, perceptions of pharmacists about cancer and breast cancer health promotion, and barriers to providing those services.^56,57,63,67–69^

A study from the UK on teledermatology indicated potential substantial cost-savings if the service was extended nationally.^44^

The evidence from the UK lacks robust clinical trials reporting on clinical outcomes of community pharmacy-based early cancer detection interventions.

### Strengths and limitations

This systematic review had a wide scope. The search strategies were comprehensive and a large number of studies were included.

Grey literature from the UK only was searched, and as a result there is potentially an omission of international non-peer-reviewed publications.

MMAT and AACODS were used for quality assessment of the included publications, to account for the difference between peer-reviewed and grey literature. The quality of the included publications was generally good. Data synthesis through meta-analysis was planned a priori; however, because of the heterogeneity of reported cancer sites, study designs, and outcomes this proved inappropriate. Subgroup analyses were planned based on the deprivation and rurality of participating pharmacies and/or service users. Rurality was included as a proxy measure of deprivation. These characteristics were, however, either not reported or were reported heterogeneously, so these analyses were not possible.

Therefore, the current review has the strength of including a wide range of evidence; however, the heterogeneity of the included publications resulted in challenges relating to presenting the data.

### Comparison with existing literature

A systematic review of community pharmacy-based education and screening interventions in 2015 identified 12 relevant studies, of which none were from the UK.^15^ Most of the included studies were from the US, and one or two studies from Italy, Australia, Germany, Spain, and Korea. The most common cancer site in the 12 publications was colorectal, and one or two breast, prostate and colorectal, prostate only, cervical only, and cervical and breast. The most common interventions were the use of a stool test and the use of risk assessments and screening questionnaires, then cervical and breast screening and prostate-specific antigen blood test. In total 52 eligible publications were identified in the current review, which may be explained by using different search strategies and inclusion criteria, with a more recent timeframe. Two papers were included in both the previous systematic review and in the current work,^31,58^ owing to fulfilling this study’s inclusion criteria. In addition, further included papers in the current work were published based on these publications.^32,60^

This current review included 16 publications from the UK, and a further 10 ongoing UK-based studies were noted. The majority of these are from the grey literature or qualitative studies. This highlights the shift in the UK from traditional pharmacy-based dispensing services to increased involvement of pharmacy staff in clinical assessment and care, which has been subject to existing UK policy.^5,6,11,92–94^ Health Education England revised the standards for the initial education and training of pharmacists in 2021, which incorporates enhanced consultation skills and clinical training.^95^

This systematic review highlights the wide range of cancer types that can be targeted by pharmacy-based programmes; however, not all may be amenable to direct referrals from pharmacies.^96^

### Implications for research and practice

Acceptability and feasibility of pharmacy-based approaches to detect cancer early have been established. However, planning such services requires multiple considerations to be taken into account, including service user and stakeholder factors, premises, digital infrastructure, and the cost–benefit ratio. These aspects should be prioritised in further international research along with robust clinical trials in the UK, summarising clinical outcomes including detection rates, stage, and diagnosis, as well as evaluating the workload implications for primary and secondary healthcare providers. Tools used for cancer risk assessment should be validated in community pharmacies.

Future research and service evaluation will require care, particularly if using routinely collected data. Data on several confounding factors will be required, such as demographics, as those presenting to community pharmacy may be different to those presenting elsewhere. Consistency of data recording in pharmacies and linking that with data from general practice and secondary care data may also pose challenges.
